# Prognostic significance of the c-erbB-2 oncogene product in childhood medulloblastoma.

**DOI:** 10.1038/bjc.1995.96

**Published:** 1995-03

**Authors:** R. J. Gilbertson, A. D. Pearson, R. H. Perry, E. Jaros, P. J. Kelly

**Affiliations:** Department of Child Health, University of Newcastle Upon Tyne Medical School, UK.

## Abstract

**Images:**


British Journal of Cancer (1995) 71, 473-477

© 1995 Stockton Press All rights reserved 0007-0920/95 $9.00

### Prognostic significance of the c-*erb*B-2 oncogene product in childhood medulloblastoma

RJ Gilbertson[1], ADJ Pearson[1], RH Perry[3], E Jaros[1] and PJ Kelly[2]

*Departments of [1]Child Health and [2]Medical Statistics, University of Newcastle Upon Tyne Medical School, Framlington Place, Newcastle Upon Tyne, UK; [3]Department of Neuropathology, Newcastle General Hospital, Westgate Road, Newcastle Upon Tyne, UK.*

**Summary** The expression and prognostic significance of the c-*erb*B-2 oncogene product was studied in 55 cases of childhood medulloblastoma. Forty-six of the 55 tumours (83.6%) expressed the c-*erb*B-2 product. The percentage of tumour cells expressing the c-*erb*B-2 product proved to be a significant indicator of patient outcome when analysed as both a categorical and a continuous variable. As a categorical variable, patients with more than 50% positive tumour cells had a significantly worse survival, with only 10% alive at 10 years *vs* 48% for those with less than 50% positive tumour cells (log rank $P = 0.0049$). To demonstrate that this observed prognostic significance was both independent and not a result of 'data-driven' categorisation, it was also entered into the Cox model as a continuous variable. Prognostic significance was retained with $P = 0.038$.

**Keywords:** medulloblastoma; prognosis; oncogene; childhood

Medulloblastoma is one of the commonest malignant tumours of the posterior fossa in children. Current surgical, and radiotherapy techniques achieve cure in between 50% and 60% of affected children (Tait *et al*., 1990). However, because the prognostic factors for this disease are not well established it has proved difficult to identify those patients who will ultimately respond to current treatment protocols. This reduces the efficient use of existing treatment regimens and the development of new therapies for non-responders.

Various clinical disease features such as patient age (Jenkin *et al*., 1990; Zerbini *et al*., 1993), sex (Bloom *et al*., 1969; Berry *et al*., 1981; Zerbini *et al*., 1993) and degree of surgical resection (Jenkin *et al*., 1990; Zerbini *et al*., 1993) have proved unreliable in predicting disease outcome. Only the presence of metastases at diagnosis (Allen and Epstein, 1982; Kopelson *et al*., 1983; Caputy *et al*., 1987; Jenkin *et al*., 1990; Zerbini *et al*., 1993) and use of posterior fossa radiotherapy dosage <50 Gy (Berry *et al*., 1981; Kopelson *et al*., 1983; Zerbini *et al*., 1993) appear constistently to indicate a poor outcome.

In recent years biological disease markers have improved the accuracy of prognostic prediction for many tumours. Moreover, the development of immunohistochemical techniques has permitted their rapid and widespread investigation. One such group of markers are oncogenes and their protein products. In the present study we investigated the expression and prognostic significance of the c-*erb*B-2 oncogene product in childhood medulloblastoma. This oncogene has previously been extensively studied in breast cancer, demonstrating a significant relationship between overexpression of its product by tumour cells and poor prognosis (Slamon *et al*., 1989; Gullick *et al*., 1991; Lovekin *et al*., 1991; Winstanley *et al*., 1991). The rodent counterpart of the c-*erb*B-2 gene, termed c-*neu*, was first identified in transplacentally induced rat neuroectodermal tumours of the central nervous system, providing evidence for the involvement of this oncogene in the development of central neuroectodermal tumours (Schecter *et al*., 1984). In addition, the commonest chromosomal abnormality in medulloblastoma is an iso-chromosome of the long arm of chromosome 17 (iso 17q) (Bigner *et al*., 1988). The c-*erb*B-2 gene is located on the long arm of chromosome 17 (Fukushige *et al*., 1986) and so is potentially involved in the abnormality.

Correspondence: RJ Gilbertson
Received 6 April 1994; revised 18 October 1994; accepted 20 October 1994

### Materials and methods

Sixty-five children less than 15 years of age with medulloblastoma were notified to the Northern Region Young People's Malignant Disease Registry between 1968 and 1988 (Craft *et al*., 1987). Four of these patients died in the post-operative period from surgical complications. Tumour material was not available for six patients. The tumours from the remaining 55 patients were studied. The age at diagnosis ranged from 1 month to 14 years with a mean of 6.3 years. Thirty-seven patients were male and 18 female. Fifty-three of the patients received a combination of surgery (total, partial or biopsy resection) with post-operative posterior fossa and craniospinal radiotherapy. Two patients were subjected to surgery alone. In addition, 24 patients received post-operative chemotherapy. In all but two this included vincristine with or without CCNU. The remaining patients received cyclophosphamide and 5-fluorouracil or 8 in 1 therapy (vincristine, cyclophosphamide, methylprednisolone, CCNU, procarbazine, cisplatin, hydroxyurea and cytosine).

The vast majority of patients had no accurate information regarding disease stage at diagnosis. This lack of data reflected both a deficiency in sensitive and routinely available imaging techniques over the period 1968-88 and the absence of a uniformly accepted method of disease staging. Therefore disease stage could not be analysed as a prognostic factor in this study.

All experimental procedures were performed using 10% formalin-fixed paraffin embedded tumour material obtained from the patients at operation. For each case, all available tumour blocks were collected for study from the Newcastle and Middlesbrough Neuropathology archives and diagnosis was confirmed by RHP.

The monoclonal antibody NCL-CB11, generated to a synthetic peptide sequence of predicted antigenicity near the C-terminus of the protein (Corbett *et al*., 1990), was used to detect the c-*erb*B-2 oncogene product in tumour sections by the avidin-biotin-peroxidase complex technique (Hsu *et al*., 1981). Five micrometre paraffin-embedded sections were cut and mounted on silanised glass slides. These were then dewaxed in xylene and rehydrated in serial alcohol solutions. Endogenous preroxidase activity was blocked by incubation in hydrogen peroxide/methanol solution followed by blockade of non-specific binding sites using 1.5% normal horse serum in Tris-buffered saline (TBS). Sections were then incubated for 16 h at 4°C in a solution of NCL CB11 monoclonal antibody (Novocastra) made up to a strength of 1:40 using 1.5% normal horse serum in TBS. Following washing in TBS, binding of the primary antibody was demonstrated with a standard avidin–biotin–peroxidase complex technique (Vectastain). This method employs a biotinylated sheep anti-mouse antibody solution followed by a colorant reaction of 0.5% diaminobenzidine and hydrogen peroxide in TBS. Sections were then counteredstained with haematoxylin.

Four controls were employed. As negative controls either primary or secondary antibodies were substituted for normal serum in the staining protocol. In addition, an antigen absorption control was performed using primary antibody first incubated with its antigen. Finally breast carcinoma tissue known to express the c-*erb*B-2 protein was employed as a positive control.

All analyses were performed blind on separate occasions by RHP and RJG. Discrepancies in staining analysis occurred in seven cases. These were re-examined on a multiheaded microscope and consensus reached. Staining was scored for three parameters: pattern of section staining, intensity of tumour cell stain and estimated percentage of section staining.

Patient survival was assessed using Kaplan–Meier survival curves and the log-rank test (Peto and Pike, 1973). Initial analysis was performed to compare the prognosis of those patients receiving chemotherapy, surgery and radiotherpy with those undergoing surgery and radiotherapy alone. No significant survival difference was observed between these two groups ($P = 0.80$). Similar analysis of patient age, sex, posterior fossa radiotherapy dose greater or less than 50 Gy and degree of surgical resection (total *vs* partial resection) for the study population also failed to reveal any prognostic significance of these variables ($P = 0.99$, 0.99, 0.23 and 0.50 respectively). The population was therefore analysed as a single group in all subsequent analysis of c-*erb*B-2 expression and survival.

Following univariate analysis the continuous variable 'percentage of positive tumour cells' was analysed in the Cox regression model with other variables including age, sex, posterior radiotherapy dose and degree of surgical resection received (Cox, 1972). This allowed the further assessment of its independent prognostic significance without the risk of 'data-driven' categorisation.

### Results

Forty-six of the 55 tumours (83.6%) expressed the c-*erb*B-2 product (Figure 1). The sparse cytoplasmic rim characteristic

**Figure 3** Medulloblastoma: focal c-*erb*B-2 product staining. Scale bar = 120 μm.

**Figure 1** Medulloblastoma: c-*erb*B-2 product cytoplasmic staining. Scale bar = 30 μm.

**Figure 4** Medulloblastoma: section demonstrating more than 50% tumour cell c-*erb*B-2 product expression. Scale bar = 15 μm.

**Figure 2** Medulloblastoma: primary antigen absorption control section. Scale bar = 30 μm.

**Figure 5** Medulloblastoma: section demonstrating less than 50% tumour cell c-*erb*B-2 product expression. Scale bar = 20 μm.

of medulloblastoma cells rendered difficult the distinction between cytoplasmic and membrane-associated c-*erb*B-2 immunoreactivity. The remaining nine (16.4%) showed no evidence of reactivity. All control sections were negative (Figure 2).

Cytoplasmic c-*erb*B-2 positivity was also seen in several normal cell types, including vascular endothelium, smooth muscle, choroid plexus epithelial cells. neurones and Purkinje cells. Staining of Purkinje cells was especially pronounced, often producting intense coarse granular cytoplasmic staining.

At low power the majority of cases revealed a heterogeneous distribution of positive tumour cells throughout the section. However, nine (19.6%) positive tumours demonstrated a focal pattern of tumour cell positivity (Figure 3). Such cases were characterised by islands of between 15 and more than 100 positive tumour cells, surrounded by large areas of faintly or non-staining tumour tissue. The distribution of foci appeared to be random, with no relationship to tumour vascularity or site within the tumour.

On a semiquantitative 1–4 scale, positive sections were scored as either 0, +, + + or + + + based on the most frequent intensity pattern observed within tumour sections. Twenty-two (47.8%) positive tumours had a predominance of intensely staining cells (+ + +), while moderate positivity (+ +) was seen in 18 (39.1%) and faint positivity (+) in the remaining six (13.1%).

The estimated percentage of tumour cells demonstrating c-*erb*B-2 product immunoreactivity within sections ranged from less than 10% to more than 80%. Twenty-three cases (50%) had an estimated section positivity of more than 50%

**Figure 6** Survival curves comparing c-*erb*B-2 product staining intensities and survival. (+), ——, (33%); (+ +), --- (48%); (+ + +), ···· (20%).

**Figure 7** Survival curves comparing focal (——, 30%) *vs* non-focal (---, 31%) c-*erb*B-2 product expression.

(Figure 4). The remaining sections expressed the c-*erb*B-2 product in fewer than 50% of cells (Figure 5).

The survival curves for the three staining variables and their respective log-rank test scores are summarised in Figures 6–8. There was no significant difference in prognosis between the three groups defined by the intensity of tumour staining (log rank $P = 0.46$). The macroscopic pattern of tumour cell c-*erb*B-2 product expression also appeared to lack prognostic significance in univariate analysis with virtually identical survival rates of 30% and 31% respectively for the two categories, focal and non-focal staining (log rank $P = 0.75$).

In contrast, the percentage of tumour cells expressing the c-*erb*B-2 product proved to be a significant indicator of patient outcome when analysed as both a categorical and continuous variable. As a categorical variable patients were divided into two groups: more or less than 50% tumour cells. The survival curve for these two categories is shown in Figure 8. Patients with more than 50% positive tumour cells had a significantly worse survival, with only 10% alive at 10 years *vs* 48% for those with less than 50% positive tumour cells (log rank $P = 0.0049$). To demonstrate that this observed prognostic significance was independent and not a result of 'data driven' categorisation, it was also entered into the Cox model as a continuous variable with other variables. These included age, sex, degree of surgical resection and posterior fossa radiotherapy dose. Only c-*erb*B-2 oncogene product expression retained prognostic significance with $P = 0.038$.

### Discussion

This study has demonstrated tumour cell expression of the c-*erb*B-2 oncogene product in a high proportion (83.6%) of childhood medulloblastomas. In addition, it reveals a significant relationship between the number of tumour cells expressing this oncogene product and patient prognosis. Patients whose tumours had c-*erb*B-2 immunoreactivity in less than 50% of tumour cells had a significantly improved 10 year survival in both log-rank and Cox analyses when compared with patients in whom more than 50% of tumour cells were positive for c-*erb*B-2 product. No significant relationship between patient survival and either the intensity of tumour cell staining or the distribution of positive cells was found. This lack of prognostic significance may relate to the small size of our study population, and further analysis is required before these two variables can be dismissed as being of no prognostic value.

The sparse rim of cytoplasm characteristic of primitive neuroectodermal tumour (PNET) cells rendered it difficult to attribute cell product immunostaining to membrane or cytop-

**Figure 8** Survival curves comparing tumours with >50% (---, 10%) and <50% (——, 48%) c-*erb*B-2 cell expression and survival.

lasm. The c-*erb*B-2 oncogene encodes a transmembrane growth factor receptor. However, both membrane and cytoplasmic expression is a well-recognised feature of c-*erb*B-2 (Gullick *et al.*, 1987; Corbett *et al.*, 1990; Winstanley *et al.*, 1991) and other members of the receptor tyrosine kinase family, including the epidermal growth factor receptor (EGFR) (Gullick *et al.*, 1991) and the more recently described c-*erb*B-3 receptor (Poller *et al.*, 1992). Although cytoplasmic immunoreactivity has been proposed to represent post-translational processing of receptor protein before membrane insertion, this remains controversial (Poller *et al.*, 1992).

In addition to tumour cell expression, specific immunostaining of several normal tissues was also demonstrated. This included heterogeneous staining of neurones and Purkinje cells, vascular endothelium and smooth muscle. Expression of c-*erb*B-2 by these normal tissues has been described in both human (Quirke *et al.*, 1989) and rat (Kokai *et al.*, 1987) fetuses. However, expression by mature human nervous tissue has not been consistently demonstrated (Natali *et al.*, 1990 Press *et al.*, 1990). In recent years various mechanisms have been proposed for the employment of antibodies directed against the c-*erb*B-2 product in the treatment of tumours expressing this protein. These include the enhancement of T-cell cytotoxicity (Shalaby *et al.*, 1992), use as immunotoxins and cytotoxin targeting or immunotherapy (Tagliabue *et al.*, 1991). Clearly, before such techniques could be considered an in-depth understanding of the expression of c-*erb*B-2 protein by normal tissues such as those described in the present study would be required.

Finally, various hypotheses have been suggested to explain the potential mechanism by which c-*erb*B-2 may initiate and promote malignant transformation. As stated earlier, the c-*erb*B-2 oncogene encodes a transmembrane growth factor receptor. Abnormalities in either the quality or quantity of c-*erb*B-2 product expressed with or without interaction with its native ligand may therefore lead to breakdown in the control of normal mitogenic signal transduction (Bargmann and Wienberg, 1988; Di Fiore *et al.*, 1990). With regard to ligand interactions, Marchionni *et al.* (1993) have recently described a family of potential ligands for the c-*erb*B-2 receptor collectively termed the neuregulins. When exposed to c-*erb*B-2-expressing cells, these proteins cause phosphorylation of the c-*erb*B-2 receptor and cell proliferation. Demonstration of neuregulin expression in the developing nervous system has led to the proposal that paracrine and autocrine processes involving c-*erb*B-2 receptor and ligand may play a key role in the development of early central nervous system tumours (Marchionni *et al.*, 1993). At the receptor level studies in human tumours have demonstrated overexpression of an otherwise normal c-*erb*B-2 product, leading to uncontrolled mitogenic signalling. One process by which overexpression may be achieved is oncogene amplification. This is principally a feature of adenocarcinomas (Yokota *et al.*, 1986) and has been described in breast (Yokota *et al.*, 1986; Berger *et al.*, 1988; Slamon *et al.*, 1989), gastric (Fukushige *et al.*, 1986; Kameda *et al.*, 1990), renal (Yokoto *et al.*, 1986) and colonic tumours (Guttman *et al.*, 1989). To date there have been no studies of the c-*erb*B-2 gene locus in medulloblastoma, and so it is unclear by what mechanism the oncogene is activated. However, the normal proto-oncogene c-*erb*B-2 maps to the long arm of chromsome 17 at q21 (Fukushige *et al.*, 1986). The principal non-random chromosomal abnormality of medulloblastoma is an iso-chromosome of the long arm chromosome 17, which can be present in multiple copies (Bigner *et al.*, 1988). This potentially results in the presence of multiple copies of the c-*erb*B-2 oncogene and hence a mechanism by which the gene may be 'amplified'. In the present study expression of the c-*erb*B-2 product was observed in 83.6% of cases, however the iso 17q abnormality is present in only 30–40% of medulloblastomas (Bigner *et al.*, 1988; Griffin *et al.*, 1988; Biegel *et al.*, 1989; Stewart *et al.*, 1990). This chromosomal abnormality is therefore unlikely to be the only potential cause of c-*erb*B-2 overexpression in this malignancy.

This study has demonstrated the expression of c-*erb*B-2 oncogene product in a high proportion of childhood medulloblastomas. In addition, the percentage of tumour cells expressing the c-*erb*B-2 product is significantly and independently related to patient prognosis. Further analysis of c-*erb*B-2 oncogene in medulloblastoma and normal nervous tissue is required to understand fully its potential role in both the pathogenesis of this malignancy and future immunotherapy.

**Acknowledgements**
Thanks are due to Dr I Corbett, Department of Pathology, University of Newcastle Upon Tyne for providing the NCl-CB11 antibody and to Dr Nurbai, Department of Pathology, Middlesbrough General Hospital, for supplying tumour material. The survival curves were generated using a program developed by J Smith and M Cole in the Department of Child Health, University of Newcastle Upon Tyne. The authors also wish to thank Mr Billy McMeekin and the staff of the Neuropathology Laboratory, Newcastle General Hospital, for their excellent technical assistance and Mrs Paula McEwen for her help in preparing this manuscript. This work was supported by the North of England Cancer Research Campaign and the North of England Children's Cancer Research Fund.

**References**

ALLEN JC AND EPSTEIN F. (1982). Medulloblastoma and other maligant neuroectodermal tumours of the CNS. The effect of patient age and extent of disease upon prognosis. *J. Neurosurg.*, **57**, 446–451.

BARGMANN CI AND WIENBERG RA. (1988). Increased tyrosine kinase activity associated with the protein encoded by the activated neu oncogene. *Proc. Natl Acad. Sci. USA*, **85**, 5394–5398.

BERGER MS, LOCHER GW, SAURER S, GULLICK WJ, WATERFIELD MD, GRONER B AND HYNES NE. (1988). Correlation of c-erb B2 gene amplification and protein expression in human breast carcinoma with nodal status and nuclear grading. *Cancer Res.*, **48**, 1238–1243.

BERRY MP, JENKIN RDT AND KEEN CW. (1981). Radiation treatment for medulloblastoma. A 21-year review. *J. Neurosurgery*, **55**, 43–51.

BIEGEL JA, RORKE LB, PACKER RJ AND EMMANUEL BS. (1989). Isochromosome 17q is the most common structural abnormality in central nervous system primitive neuroectodermal tumours. *Paediatr. Neurosci.*, **14**, 150–160.

BIGNER SH, MARK J, FRIEDMAN HS, BIEGEL JA AND BIGNER DD. (1988). Structural chromosomal abnormalities in human medulloblastomas. *Cancer Genet. Cytogenet.*, **30**, 91–101.

BLOOM HJG, WALLACE ENK AND HENK JM. (1969). The treatment and prognosis of medulloblastoma in children: a study of 82 verified cases. *Am. J. Radiol.*, **105**, 43–62.

CAPUTY AJ, McCULLOUGH DC, HERBERT MJ, PATTERSON K AND HAMMOCK MK. (1987). A review of the factors influencing the prognosis of medulloblastoma. The importance of cell differentiation. *J. Neurosurg.*, **66**, 80–87.

CORBETT IP, HENRY JA, ANGUS B, WATCHORN CJ, WILKINSON L, HENNESY C, GULLICK WJ, TUZI NL, MAY FEB, WESTLEY BR AND HORNE CHW. (1990). NCL-CB11, a new monoclonal antibody recognising the internal domain of the c-erb B2 oncogene protein effective for use on formalin-fixed paraffin embedded tissue. *J. Pathol.*, **161**, 15–25.

COX DR. (1972). Regression models and life tables. *J. R. Stat. Soc. B.*, **34**, 187–220.

CRAFT AW, AMINEDDINE HA, SCOTT JES AND WAGGET J. (1987). The Northern Region Children's malignant disease registry 1968–1982: incidence and survival. *Br. J. Cancer*, **56**, 853–858.

DI FIORE PP, SEGATTO O, LONARDO F, FAZIOLI F, PIERCE JH AND AARONSON S. (1990). The carboxy terminal domains of c-erb B2 and EGFR exert different regulatory effects on intrinsic receptor tyrosine kinase function and transforming activity. *Mol. Cell. Biol.*, **10**, 2749–2756.

FUKUSHIGE SI, MATSUBARA KI, YOSHIDA M, SASAKI M, SUZUKI T, SEMBA K, TOYOSHIMA K AND YAMAMOTO T. (1986). Localisation of a novel v-erb B related gene c-erb B2 on human chromosome 17 and its amplification in a gastric cancer cell line. *Mol. Cell. Biol.*, **6**, 955–958.

GRIFFIN CA, HAWKINS AL, PACKER RJ, RORKE LB AND EMANUEL BS. (1988). Chromosome abnormalities in paediatric brain tumours. *Cancer Res.*, **48**, 175–180.

GULLICK WJ, BERGER MS, BENNETT PLP, ROTHBARD JB AND WATERFIELD MD. (1987). Expression of the c-erb B2 protein in normal and transformed cells. *Int. J. Cancer*, **40**, 246–254.

GULLICK WJ, LOVE SB, WRIGHT C, BARNES DM, GUSTERSON B, HARRIS AL AND ALTMAN DG. (1991). C-erb B2 overexpression in breast cancer is a risk factor in patients with involved and uninvolved lymph nodes. *Br. J. Cancer*, **63**, 434–438.

GUTMAN M, RAVIA Y, ASSAF D, YAMAMOTO T, ROZIN R AND SHILOH Y. (1989). Amplification of c-myc and c-erb B2 proto-oncogenes in human solid tumours: frequency and clinical signficance. *Int. J. Cancer*, **44**, 802–805.

HSU SM, RAINE L AND FANGER H. (1981). Use of avidin–biotin peroxidase complex (ABC) in immunoperoxidase techniques: a comparison between ABC and unlabelled antibody (PAP) procedures. *J. Histochem. Cytochem.*, **29**, 577–580.

HUDZIAK RM, LEWIS GD, WINGET M, FENDLY BM, SHEPARD M AND ULLRICH A. (1989). P185HER-2 monoclonal antibody has antiproliferative effects in vitro and sensitises human breast cancer cells to tumour necrosis factor. *Mol. Cell. Biol.*, **9**, 1165–1172.

JENKIN D, GODDARD K, ARMSTRONG D, BECKER L, BERRY MP, CHAN H, DOHERTY M, GREENBERG M, HENDRICK B, HOFFMAN H, HUMPHREYS H, SENLEY M, WIETZMAN S AND ZIPERSKY A. (1990). Posteria fossa medulloblastoma in childhood. Treatment results and a proposal of a new staging system. *Int. J. Radiat. Oncol. Biol. Phys.*, **19**, 265–274.

KAMEDA T, YASUI W, YOSHIDA K, TSUJINO T, NAKAYAMA H, ITO H AND TAHARA E. (1990). Expression of ERB B2 in human gastric carcinomas: relationship between pl85 expression and the gene amplification. *Cancer Res.*, **50**, 8002–8009.

KOKAI Y, COHEN JA, DREBIN JA AND GREENE MI. (1987). Stage and tissue specific expression of the nru oncogene in rat development. *Proc. Natl Acad. Sci. USA*, **84**, 8498–8501.

KOPELSON G, LINGGOOD RM AND KLEINMAN GM. (1983). Medulloblastoma. The identification of prognostic subgroups and implications for multimodality treatment. *Cancer*, **51**, 312–319.

LOVEKIN C, ELLIS IO, LOCKER A, ROBERTSON JFR, BELL J, NICHOLSON R, GULLICK WJ, ELESTON CW AND BLANEY RW. (1991). C-erb B2 oncoprotein expression in primary and advanced breast cancer. *Br. J. Cancer*, **63**, 439–443.

MARCHIONNI MA, GOODEARL ADJ, MAIO SC, BERMINGHAM-McDONOGH O, KIRK C, HENDRICKS M, DANEHY F, MISUMI D, SUDHALTER J, KOBAYASHI K, WROBLEWSKI D, LYNCH C, BALDASSARE M, HILES I, DAVIS JB, HSUAN JJ, TOTTY NF, OTSU M, McBURNEY RN, WATERFIELD MD, STROOBANT P AND GWYNNE D. (1993). Glial growth factors are alternatively spliced c-erb B2 ligands expressed in the nervous system. *Nature*, **362**, 312–318.

NATALI PG, NICOTRA MR, BIGOTTI A, VENTURO I, SLAMON DJ, FENDLY BM AND ULLRICH A. (1990). Expression of the pl85 encoded by the HER 2 oncogene in normal and transformed human tissues. *Int. J. Cancer*, **45**, 457–461.

PETO R AND PIKE MC. (1973). Conservatism in the approximation E (O-E) 2/E in the log rank tests for survival data and tumour incidence data. *Biometrics*, **29**, 579–584.

POLLER DN, SPENDLOVE I, BAKER C, CHURCH R, ELLIS IO, PLOWMAN GD AND MAYER RJ (1992). Production and characterisation of a polyclonal antibody to the c-erbB-3 protein: examination of c-erbB-3 protein expression in adenocarcinomas. *J. Pathol.*, **168**, 275–280.

PRESS MF, CORDON-CARDER C AND SLAMON D. (1990). Expression of the HER-2/neu proto-oncogene in normal adult and fetal tissues. *Oncogene*, **5**, 935–962.

QUIRKE P, PICKLES A, TUZI NL, MOHAMDEE O AND GULLICK WJ. (1989). Pattern of expression of c-erb B2 onco-protein in human fetuses. *Br. J. Cancer*, **60**, 64–69.

SCHECTER A, STERN D, VAIDYANATHAN L, DECTER S, DREBIN J, GREENE M AND WIENBERG RA. (1984). The neu-oncogene: an erb B-related gene encoding a 185,000 MR tumour antigen. *Nature*, **312**, 513–516.

SHALABY MR, SHEPARD HM, PRESTA L, RODRIGUES ML, BEVERLEY PCL, FELDMANN M AND CARTER P. (1992). Development of humanized biospecific antibodies reactive with cytotoxic lymphocytes and tumour cells overexpressing the HER2 protoncogene. *J. Exp. Med.*, **175**, 217–223.

SLAMON DJ, GODOLPHIN W, JONES LA AND WANG SG. (1989). Studies of the HER-2/neu proto-oncogene in human breast and ovarian cancer. *Science*, **244**, 707–712.

STEWART AG, PEARSON ADJ, EMSLIE J, LENNARD A, DAVISION EV, PERRY RH AND CRAWFORD PJ. (1990). Cytogenic abnormalities in disseminated medulloblastoma. *J. Med. Paediatr. Oncol.*, **18**, 170–176.

TAIT DM, THORNTON-JONES H, BLOOM HJG, LEMERLE J AND MORRIS-JONES P. (1990). Adjuvant chemotherapy for medulloblastoma: the first multicentre control trial of the International Society of Paediatric Oncology (SIOPI). *Eur. J. Cancer*, **26**, 464–469.

TAGLIABUE E, CENTI F, CAMPIGLIO M, MARTIGNONE S, PELLEGINI R, CASALINI P, LANZI C, MENARD S, COLAGHI MI. (1991). Selection of monoclonal antibodies which induce internalisation and phosphorylation of pl185HER2 and growth inhibition of cells with HER2/neu gene amplification. *Int. J. Cancer*, **47**, 933–937.

WINSTANLEY J, COOKE T, MURRAY GD, PLATT-HIGGINS A, GEORGE WD, HOLT S, MYZKOV M, SPEDDING A, BARRACLOUGH BR AND RUDLAND PS. (1991). The long-term prognostic significance of c-erb B2 in primary breast cancer. *Br. J. Cancer*, **63**, 447–450.

YOKOTA J, YAMAMOTO T, TOYOSHIMA K, TERADA M, SUJEMIRA T AND BATTIFORA H. (1986). Amplification of c-erb B2 oncogene in human adenocarcinomas *in vivo*. *Lancet*, **i**, 765–766.

ZERBINI C, GELBER RD, WIENBERG D, SALLAN SE, BARNES P, KUPSKY W, SCOTT M AND TARBELL NJ. (1993). Prognostic factors in medulloblastoma including DNA ploidy. *J. Clin. Oncol.*, **11**, 616–622.


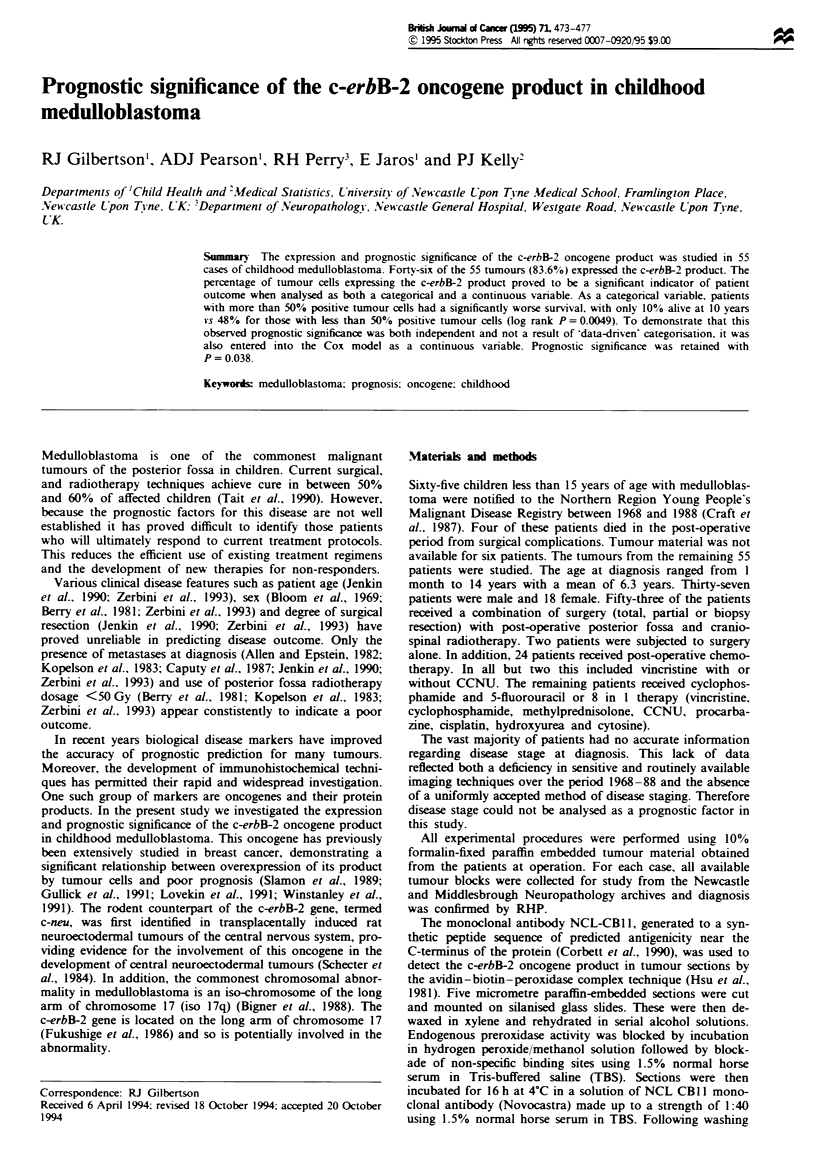

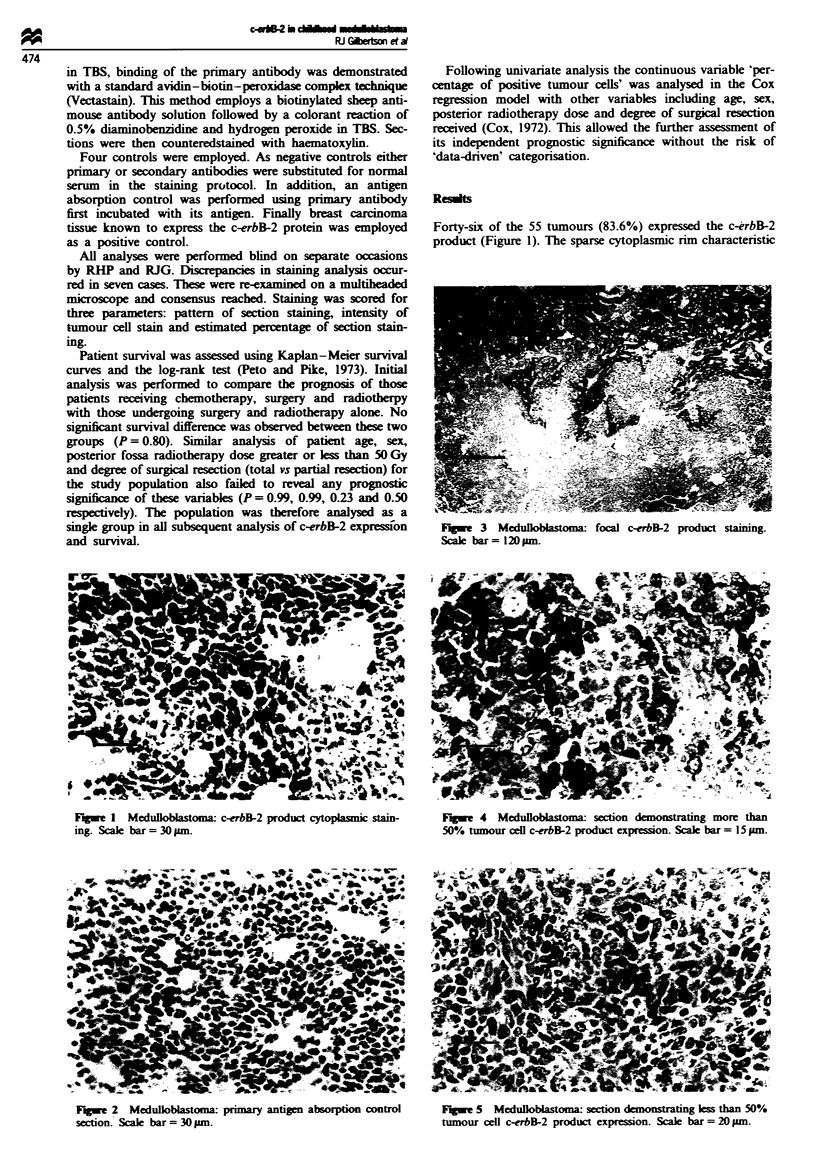

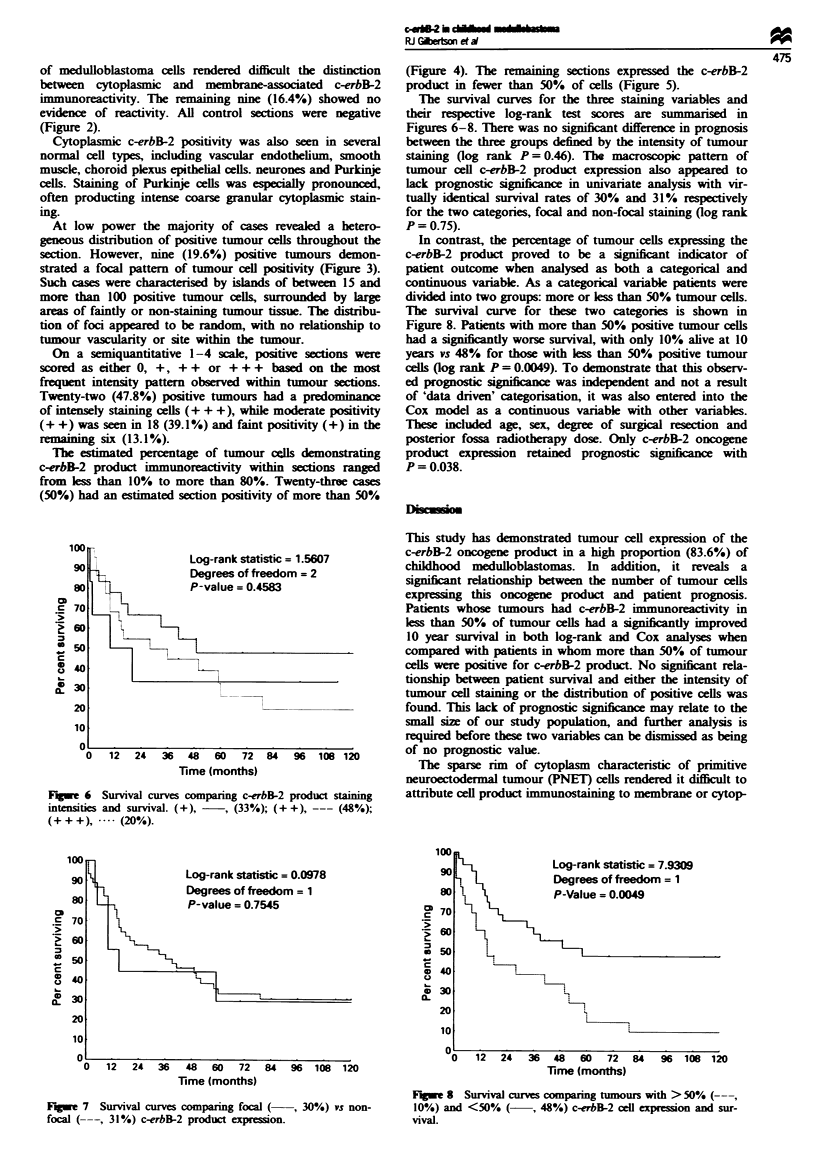

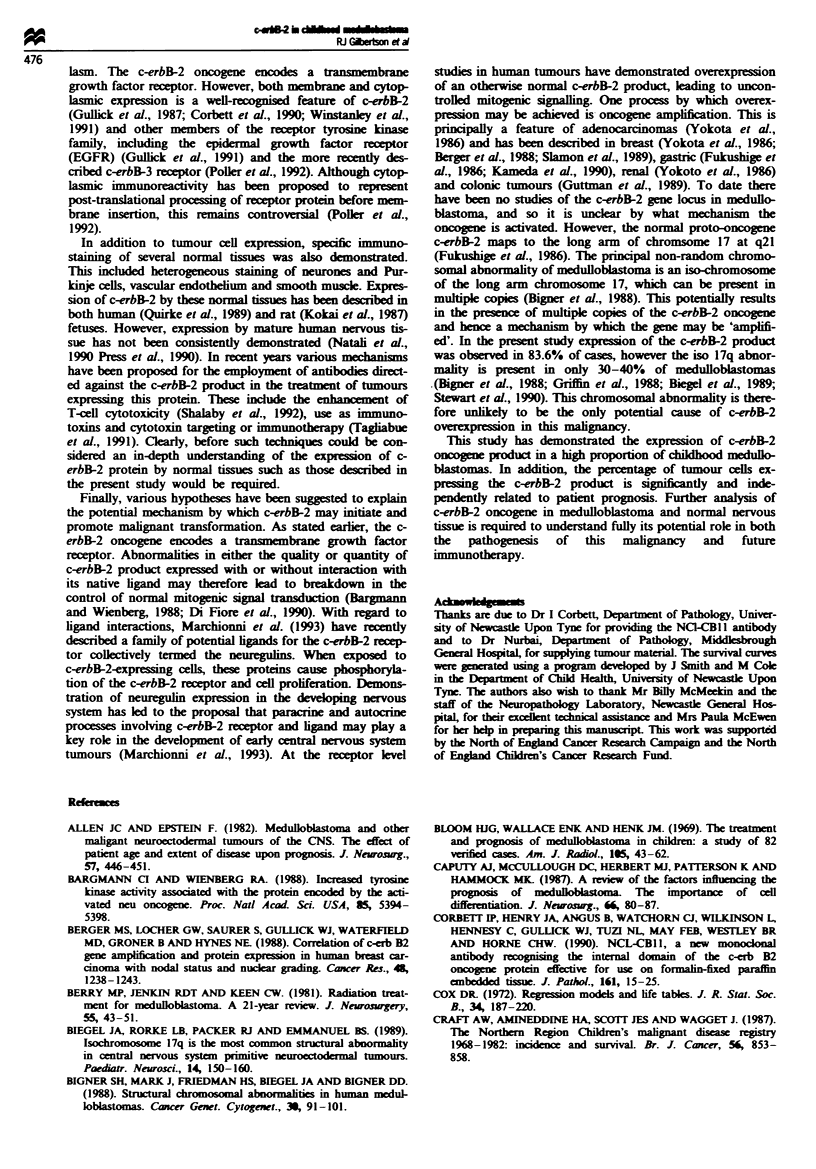

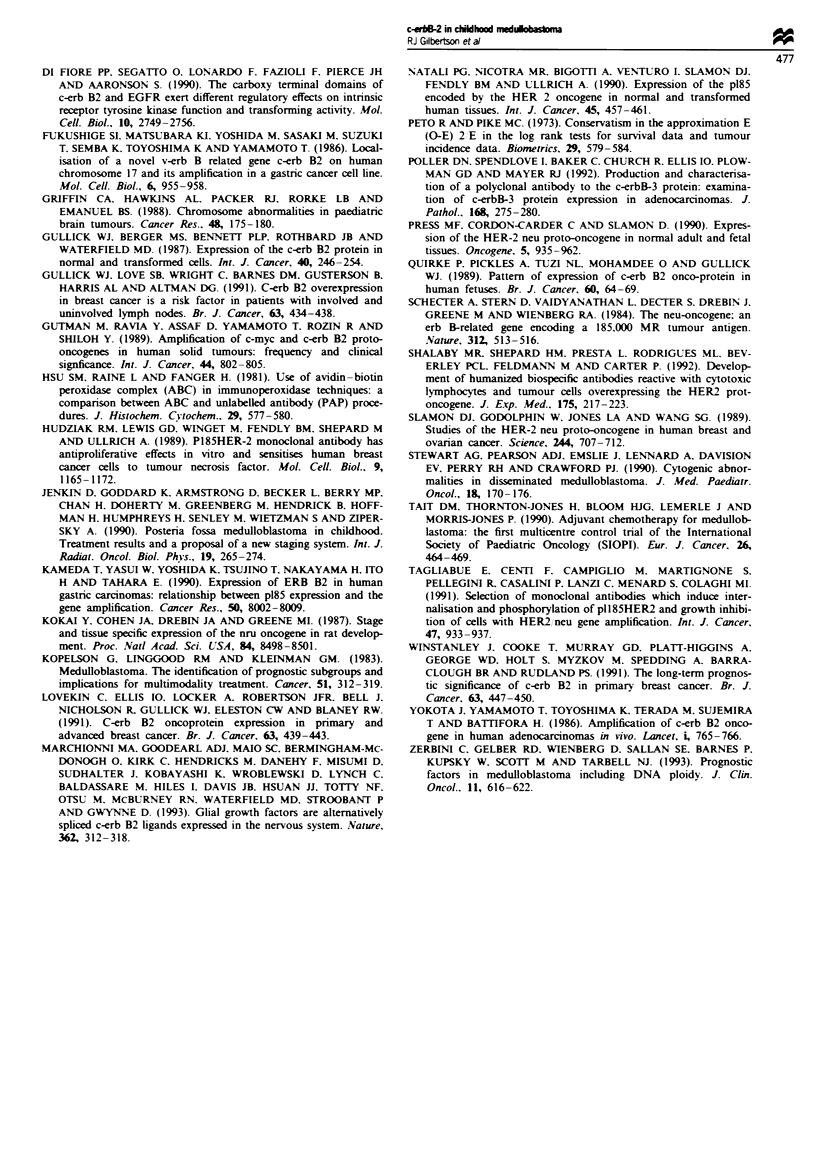


## References

[OCR_00504] Allen J. C., Epstein F. (1982). Medulloblastoma and other primary malignant neuroectodermal tumors of the CNS. The effect of patients' age and extent of disease on prognosis.. J Neurosurg.

[OCR_00510] Bargmann C. I., Weinberg R. A. (1988). Increased tyrosine kinase activity associated with the protein encoded by the activated neu oncogene.. Proc Natl Acad Sci U S A.

[OCR_00516] Berger M. S., Locher G. W., Saurer S., Gullick W. J., Waterfield M. D., Groner B., Hynes N. E. (1988). Correlation of c-erbB-2 gene amplification and protein expression in human breast carcinoma with nodal status and nuclear grading.. Cancer Res.

[OCR_00521] Berry M. P., Jenkin R. D., Keen C. W., Nair B. D., Simpson W. J. (1981). Radiation treatment for medulloblastoma. A 21-year review.. J Neurosurg.

[OCR_00532] Bigner S. H., Mark J., Friedman H. S., Biegel J. A., Bigner D. D. (1988). Structural chromosomal abnormalities in human medulloblastoma.. Cancer Genet Cytogenet.

[OCR_00542] Caputy A. J., McCullough D. C., Manz H. J., Patterson K., Hammock M. K. (1987). A review of the factors influencing the prognosis of medulloblastoma. The importance of cell differentiation.. J Neurosurg.

[OCR_00550] Corbett I. P., Henry J. A., Angus B., Watchorn C. J., Wilkinson L., Hennessy C., Gullick W. J., Tuzi N. L., May F. E., Westley B. R. (1990). NCL-CB11, a new monoclonal antibody recognizing the internal domain of the c-erbB-2 oncogene protein effective for use on formalin-fixed, paraffin-embedded tissue.. J Pathol.

[OCR_00562] Craft A. W., Amineddine H. A., Scott J. E., Wagget J. (1987). The Northern region Children's malignant disease registry 1968-82: incidence and survival.. Br J Cancer.

[OCR_00572] Di Fiore P. P., Segatto O., Lonardo F., Fazioli F., Pierce J. H., Aaronson S. A. (1990). The carboxy-terminal domains of erbB-2 and epidermal growth factor receptor exert different regulatory effects on intrinsic receptor tyrosine kinase function and transforming activity.. Mol Cell Biol.

[OCR_00582] Fukushige S., Matsubara K., Yoshida M., Sasaki M., Suzuki T., Semba K., Toyoshima K., Yamamoto T. (1986). Localization of a novel v-erbB-related gene, c-erbB-2, on human chromosome 17 and its amplification in a gastric cancer cell line.. Mol Cell Biol.

[OCR_00586] Griffin C. A., Hawkins A. L., Packer R. J., Rorke L. B., Emanuel B. S. (1988). Chromosome abnormalities in pediatric brain tumors.. Cancer Res.

[OCR_00594] Gullick W. J., Berger M. S., Bennett P. L., Rothbard J. B., Waterfield M. D. (1987). Expression of the c-erbB-2 protein in normal and transformed cells.. Int J Cancer.

[OCR_00598] Gullick W. J., Love S. B., Wright C., Barnes D. M., Gusterson B., Harris A. L., Altman D. G. (1991). c-erbB-2 protein overexpression in breast cancer is a risk factor in patients with involved and uninvolved lymph nodes.. Br J Cancer.

[OCR_00605] Gutman M., Ravia Y., Assaf D., Yamamoto T., Rozin R., Shiloh Y. (1989). Amplification of c-myc and c-erbB-2 proto-oncogenes in human solid tumors: frequency and clinical significance.. Int J Cancer.

[OCR_00610] Hsu S. M., Raine L., Fanger H. (1981). Use of avidin-biotin-peroxidase complex (ABC) in immunoperoxidase techniques: a comparison between ABC and unlabeled antibody (PAP) procedures.. J Histochem Cytochem.

[OCR_00617] Hudziak R. M., Lewis G. D., Winget M., Fendly B. M., Shepard H. M., Ullrich A. (1989). p185HER2 monoclonal antibody has antiproliferative effects in vitro and sensitizes human breast tumor cells to tumor necrosis factor.. Mol Cell Biol.

[OCR_00621] Jenkin D., Goddard K., Armstrong D., Becker L., Berry M., Chan H., Doherty M., Greenberg M., Hendrick B., Hoffman H. (1990). Posterior fossa medulloblastoma in childhood: treatment results and a proposal for a new staging system.. Int J Radiat Oncol Biol Phys.

[OCR_00632] Kameda T., Yasui W., Yoshida K., Tsujino T., Nakayama H., Ito M., Ito H., Tahara E. (1990). Expression of ERBB2 in human gastric carcinomas: relationship between p185ERBB2 expression and the gene amplification.. Cancer Res.

[OCR_00637] Kokai Y., Cohen J. A., Drebin J. A., Greene M. I. (1987). Stage- and tissue-specific expression of the neu oncogene in rat development.. Proc Natl Acad Sci U S A.

[OCR_00642] Kopelson G., Linggood R. M., Kleinman G. M. (1983). Medulloblastoma. The identification of prognostic subgroups and implications for multimodality management.. Cancer.

[OCR_00644] Lovekin C., Ellis I. O., Locker A., Robertson J. F., Bell J., Nicholson R., Gullick W. J., Elston C. W., Blamey R. W. (1991). c-erbB-2 oncoprotein expression in primary and advanced breast cancer.. Br J Cancer.

[OCR_00662] Natali P. G., Nicotra M. R., Bigotti A., Venturo I., Slamon D. J., Fendly B. M., Ullrich A. (1990). Expression of the p185 encoded by HER2 oncogene in normal and transformed human tissues.. Int J Cancer.

[OCR_00668] Peto R., Pike M. C. (1973). Conservatism of the approximation sigma (O-E)2-E in the logrank test for survival data or tumor incidence data.. Biometrics.

[OCR_00674] Poller D. N., Spendlove I., Baker C., Church R., Ellis I. O., Plowman G. D., Mayer R. J. (1992). Production and characterization of a polyclonal antibody to the c-erbB-3 protein: examination of c-erbB-3 protein expression in adenocarcinomas.. J Pathol.

[OCR_00685] Quirke P., Pickles A., Tuzi N. L., Mohamdee O., Gullick W. J. (1989). Pattern of expression of c-erbB-2 oncoprotein in human fetuses.. Br J Cancer.

[OCR_00690] Schechter A. L., Stern D. F., Vaidyanathan L., Decker S. J., Drebin J. A., Greene M. I., Weinberg R. A. (1984). The neu oncogene: an erb-B-related gene encoding a 185,000-Mr tumour antigen.. Nature.

[OCR_00696] Shalaby M. R., Shepard H. M., Presta L., Rodrigues M. L., Beverley P. C., Feldmann M., Carter P. (1992). Development of humanized bispecific antibodies reactive with cytotoxic lymphocytes and tumor cells overexpressing the HER2 protooncogene.. J Exp Med.

[OCR_00701] Slamon D. J., Godolphin W., Jones L. A., Holt J. A., Wong S. G., Keith D. E., Levin W. J., Stuart S. G., Udove J., Ullrich A. (1989). Studies of the HER-2/neu proto-oncogene in human breast and ovarian cancer.. Science.

[OCR_00722] Tagliabue E., Centis F., Campiglio M., Mastroianni A., Martignone S., Pellegrini R., Casalini P., Lanzi C., Ménard S., Colnaghi M. I. (1991). Selection of monoclonal antibodies which induce internalization and phosphorylation of p185HER2 and growth inhibition of cells with HER2/NEU gene amplification.. Int J Cancer.

[OCR_00714] Tait D. M., Thornton-Jones H., Bloom H. J., Lemerle J., Morris-Jones P. (1990). Adjuvant chemotherapy for medulloblastoma: the first multi-centre control trial of the International Society of Paediatric Oncology (SIOP I).. Eur J Cancer.

[OCR_00729] Winstanley J., Cooke T., Murray G. D., Platt-Higgins A., George W. D., Holt S., Myskov M., Spedding A., Barraclough B. R., Rudland P. S. (1991). The long term prognostic significance of c-erbB-2 in primary breast cancer.. Br J Cancer.

[OCR_00734] Yokota J., Yamamoto T., Toyoshima K., Terada M., Sugimura T., Battifora H., Cline M. J. (1986). Amplification of c-erbB-2 oncogene in human adenocarcinomas in vivo.. Lancet.

[OCR_00739] Zerbini C., Gelber R. D., Weinberg D., Sallan S. E., Barnes P., Kupsky W., Scott R. M., Tarbell N. J. (1993). Prognostic factors in medulloblastoma, including DNA ploidy.. J Clin Oncol.

